# Stage-Specific MicroRNAs and Their Role in the Anticancer Effects of Calorie Restriction in a Rat Model of ER-Positive Luminal Breast Cancer

**DOI:** 10.1371/journal.pone.0159686

**Published:** 2016-07-19

**Authors:** Kaylyn L. Devlin, Tiffany Sanford, Lauren M. Harrison, Paul LeBourgeois, Laura M. Lashinger, Elizabeth Mambo, Stephen D. Hursting

**Affiliations:** 1 Department of Molecular Biosciences, The University of Texas at Austin, Austin, Texas, United States of America; 2 Asuragen Incorporated, Austin, Texas, United States of America; 3 Department of Nutritional Sciences, The University of Texas at Austin, Austin, Texas, United States of America; National Institutes of Health, UNITED STATES

## Abstract

MicroRNAs have emerged as ubiquitous post-transcriptional regulators that coordinate many fundamental processes within cells, including those commonly linked to cancer when dysregulated. Profiling microRNAs across stages of cancer progression provides focus as to which microRNAs are key players in cancer development and are therefore important to manipulate with interventions to delay cancer onset and progression. Calorie restriction is one of the most effective preventive interventions across many types of cancer, although its effects on microRNAs have not been well characterized. We used the dimethylbenz[a]-anthracene-induced model of luminal mammary cancer in Sprague Dawley rats to elucidate which microRNAs are linked to progression in this type of cancer and, subsequently, to study how calorie restriction affects such microRNAs. We identified eight microRNAs (miR-10a, miR-10b, miR-21, miR-124, miR-125b, miR-126, miR-145 and miR-200a) to be associated with DMBA-induced mammary tumor progression. Calorie restriction, which greatly increased tumor-free survival and decreased the overall size of tumors that did develop, significantly decreased the expression of one microRNA, miR-200a, which was positively associated with tumor progression. We further showed that inhibition of miR-200a function, mimicking the effect of calorie restriction on this microRNA, inhibited proliferation in both rat (LA7) and human (MCF7) luminal mammary cancer cell lines. These findings present, for the first time, a stage-specific profile of microRNAs in a rodent model of luminal mammary cancer. Furthermore, we have identified the regulation of miR-200a, a microRNA that is positively associated with progression in this model, as a possible mechanism contributing to the anticancer effects of calorie restriction.

## Introduction

Calorie restriction (CR), a dietary regimen in which subjects receive a nutritionally replete but reduced energy diet (typically ~30% reduction in total energy intake) is arguably the most potent and broadly acting dietary intervention for preventing or inhibiting cancer in experimental tumor models, including rodent models of several intrinsic subtypes of breast cancer [1]. Moreover, CR is increasingly being applied to human cancer as a preventive strategy or in cancer patients as a sensitizing strategy prior to chemotherapeutic or radiation therapy regimens [2,3,4]. While the beneficial effects of CR are well established, the mechanisms through which CR affects cancer are poorly understood, hampering efforts to translate CR to the prevention and control of human cancer [5,6].

A broad spectrum of genes are modulated by CR, suggesting that this dietary intervention may modulate one or more master regulators of gene expression [7]. One such regulation system functions through microRNAs (miRNAs), which are small (21–25 nucleotide) non-protein coding RNAs known to broadly regulate the expression and/or translation of mRNAs [8]. Breast cancer has been a major focus of miRNA research over the past decade, leading to the elucidation of the oncogenic or tumor suppressive functions of miRNAs via regulation of target mRNAs involved in several breast cancer hallmarks, including tumor growth, apoptosis, invasion, and inflammation [9,10]. Advances in miRNA profiling have aided in the discovery of these key cancer regulators and are facilitating the definition of miRNA expression patterns across different tissue types [11]. Profiling of human breast samples has identified differentially expressed miRNAs in many important comparisons, including between normal and cancerous tissue, during the progression stages leading up to invasive disease, between tumor subtypes, and between cases with varying clinical outcomes [11,12,13,14,15,16]. However, no profiling to date has focused on the development and progression of breast cancer within an individual subtype, which would provide a more accurate assessment of miRNA signatures considering there are significant subtype-specific differences in miRNA expression. Furthermore, the potential impact of dietary energy balance modulation, such as CR, on progression-related miRNA profiles has not yet been characterized.

Here, we used the dimethylbenz[a]-anthracene (DMBA)-induced mammary cancer model in Sprague Dawley rats and global miRNA expression array analysis to profile miRNA expression across multiple stages of luminal mammary tumor development and progression. The DMBA rat model was selected since DMBA induces preneoplastic lesions, including intraductal proliferation (IDP) and mammary intraepithelial neoplasias (MIN, equivalent to ductal carcinoma in situ in humans) that progress to ER-positive invasive ductal carcinomas (IDC) in rats that are similar to the most prevalent subtype of breast cancer (luminal A) in women [17,18,19,20]. We also tested the hypothesis that CR inhibits mammary tumor development and/or progression in association with alterations in one or more key miRNAs identified in our profiling studies. We identified, for the first time to our knowledge, a panel of miRNAs associated with luminal mammary tumor progression. Moreover, we found that CR strongly suppresses DMBA-induced mammary tumor development, and that one of these progression-associated miRNAs, miR-200a, is highly responsive to CR and may be an important contributor to the anticancer effects of CR.

## Materials and Methods

### Rats, diets, and study design

All rats were housed in the Animal Resource Center at the University of Texas at Austin. All animal studies and procedures were approved and monitored by the Institutional Animal Care and Use Committee at the University of Texas. Control rats were monitored at least once per week, while CR rats were monitored daily and given nestlets and huts to alleviate distress. No rats died prior to study endpoints.

Rats were purchased from Charles River Laboratories, Wilmington, MA. Upon arrival, 208 5-week old female Sprague Dawley rats, housed 3 per cage, received a modified AIN-76A diet (control, consumed *ad libitum*; catalog # D12450B, Research Diets, Inc., New Brunswick, NJ) for two weeks. After this acclimation period, rats were randomly assigned to receive a single dose via gavage of either a) the carcinogen dimethylbenz(a)anthracene (DMBA) (65mg/kg, dissolved in sesame oil [30mg/mL], n = 155) or b) sesame oil alone (vehicle, equivalent volume, n = 53). Rats were further randomized into one of two substudies: a) a time course study (DMBA, n = 95; vehicle, n = 13) or b) a tumor-free survival study including dietary intervention (DMBA, n = 60; vehicle, n = 40).

#### Time course study

Beginning six weeks after DMBA administration, and continuing until study endpoint (16 weeks post-DMBA administration), rats were palpated every two weeks and euthanized when tumor was first discovered for mammary gland collection. Vehicle-treated rats had no tumor development and were euthanized 16 weeks after DMBA treatment (study endpoint). All rats were fasted for 12 hours, anesthetized with CO_2_, and then euthanized by cervical dislocation.

#### Tumor-free survival study

Rats assigned to the survival substudy were singly housed and randomized to receive either a) control diet, (described above; DMBA, n = 20; vehicle, n = 20) or b) a 30% calorie restriction diet (CR; consumed as daily aliquot; DMBA, n = 40; vehicle, n = 20; catalog # D03020702, Research Diets, Inc). The CR diet is a modified AIN-76A semi-purified diet that is calculated to provide 30% less total calories while maintaining 100% of all vitamins, minerals, fatty acids, and amino acids relative to the control group. Mammary fat pads were palpated twice weekly, and rats were euthanized as previously described if one of two criteria were met: a tumor reached 1.2 cm in any direction or study endpoint was reached (12 weeks post-DMBA administration). The number of rats in the CR diet group was higher than in the control diet group to ensure that, in the event CR inhibited tumor development, we would collect sufficient tumor material from rats on the CR diet for subsequent analyses.

### Tissue processing and histopathologic analysis

Mammary fat pad and tumor tissue was excised, split longitudinally, and either snap-frozen in liquid nitrogen and stored at -80°C or fixed in 10% neutral-buffered formalin for 24 hours, transferred to 70% ethanol for 24 hours, and embedded in paraffin. Formalin-fixed, paraffin-embedded (FFPE) mammary fat pads were cut into 4-μm sections, placed on slides, deparaffinized in xylene, and rehydrated. Slides were stained with hematoxylin and eosin (H&E) and histologically assessed (in a blinded manner) by a board-certified pathologist (P. LeBourgeois) into one of four stages of cancer progression: normal tissue, intraductal proliferation (IDP), mammary intraepithelial neoplasia (MIN), or invasive ductal carcinoma (IDC) [20].

### MicroRNA expression analysis

#### RNA extraction

FFPE tissue from the time course study was microdissected in a progression stage-specific manner so that comparisons between different stages could be conducted. Once tissue was microdissected, total RNA was isolated using RecoverAll^™^ Total Nucleic Acid Isolation Kit for FFPE (Ambion, Carlsbad, CA) following manufacturer’s protocols. Total RNA from the survival study was isolated from flash-frozen tumors using TRI Reagent^®^ (Sigma-Aldrich, St. Louis, MO) according to manufacturer’s instructions. RNA quantity and quality was measured on a Nanodrop 2000 spectrophotometer (Thermo Scientific, Waltham, MA) and a 2100 Bioanalyzer (Agilent Technologies, Santa Clara, CA).

#### Time course study microRNA array and validation

Total RNA samples from cancer stage-specific lesions (n = 2/stage) from the time course study were run on an Affymetrix GeneChip miRNA 2.0 array (Affymetrix, Santa Clara, CA) at Asuragen, Inc. (Austin, TX) as previously described (Ovcharenko 2011). The following criteria were used to select miRNAs of interest that were trending toward a relevant effect in this preliminary screen: an average fluorescence that was 50% above minimal acceptable limit and at least a two-fold change in expression between any two stages.

MicroRNAs that were selected in this manner were then extensively analyzed in several reverse transcription quantitative PCR-based (RT-qPCR) validation steps. Complementary DNA of miRNAs for PCR-based analyses was generated using the Universal cDNA Synthesis Kit (Exiqon, Vedbaek, Denmark). Stage I validation included all array-identified miRNAs, in addition to some well-established breast cancer-associated miRNAs (44 miRNAs in total). Stage I validation was done using the same RNA samples run on the array (n = 2/stage) with a custom Pick-&-Mix microRNA PCR Panel (each probe-sample combination plated in duplicate) and SYBR^®^ Green master mix (Exiqon) on a Mastercycler^®^ ep gradient S (Eppendorf, Hamburg, Germany). The miRNAs from stage I validation identified as trending toward a relevant effect (fold change between two stages of at least 2.0) were included in stage II validation (15 miRNAs total). Stage II validation analyzed the expression of these 15 miRNAs in 6 freshly-extracted total-RNA samples per stage by RT-qPCR using microRNA LNA^™^ PCR primer sets (plated in triplicate) and SYBR^®^ Green master mix (Exiqon) on a ViiA^™^ 7 Real-Time PCR system (Applied Biosystems, Carlsbad, CA). MicroRNA-16 was used as the reference gene for PCR-based analyses and relative expression calculations were done using the ΔΔCt method.

#### Survival study RT-qPCR analyses

RNA extracted from tumors from control and CR-fed rats was analyzed for expression of miRNAs identified in the time course study. Complementary DNA of miRNAs for these analyses was generated using the TaqMan^®^ MicroRNA Reverse Transcription Kit (Applied Biosystems). Individual miRNA assays were done using TaqMan^®^ MicroRNA Assays and TaqMan^®^ Universal PCR Master Mix on a ViiA^™^ 7 Real-Time PCR system (Applied Biosystems). All steps of assays were done according to manufacturer’s protocols. MicroRNA-16 was used as the reference gene for all PCR-based analyses and relative expression calculations were done using the ΔΔCt method.

### MiR-200a inhibition and proliferation assays

Rat LA7 cells (obtained directly from ATCC, catalog #: CRL-2283), which were originally derived from a DMBA-induced mammary tumor in a Sprague Dawley rat, and human MCF7 cells (obtained directly from ATCC, catalog #: HTB-22) were used in *in vitro* experiments. MicroRNA-200a inhibition was performed using 5nM miR-200a-targeting and non-targeting control power inhibitors (Exiqon), transfected in tandem with a synthetic miR-200a target RenSP luciferase reporter plasmid (Switchgear Genomics, Carlsbad, CA) at a ratio of 1:5 (ng:nL) with Dharmafect Duo transfection reagent (GE Healthcare, Pittsburgh, PA). Twenty-four hours after transfection, cells were seeded into two separate 96-well plates, one for luciferase readout to verify miRNA inhibition and one for proliferation analysis. Luciferase and proliferation analyses were done 24 hours after reseeding, for a total of 48 hours after miRNA inhibition. RenSP luciferase signal was analyzed using LightSwitch Assay Reagent (Switchgear Genomics) according to manufacturer protocol. Cellular proliferation was measured using a BrdU ELISA kit (Roche, Basel, Switzerland) following manufacturer protocol.

### Statistical analyses

Summarized data are reported as mean ± SEM, except body weight and tumor area data which are reported as mean ± SD. Analyses and statistics of array data were done as previously described [21]. Tumor area difference was tested using a Mann Whitney U test. Survival curves were compared using the log-rank test, and body weight was analyzed at 9 weeks on diet using one-way ANOVA. Differences in miRNA expression and luciferase signal were assessed using the unpaired, two-tailed Student’s t-test. Cellular proliferation analysis was done using a one-sample t-test. Significance was determined at P < 0.05.

## Results

**Fig 1:**
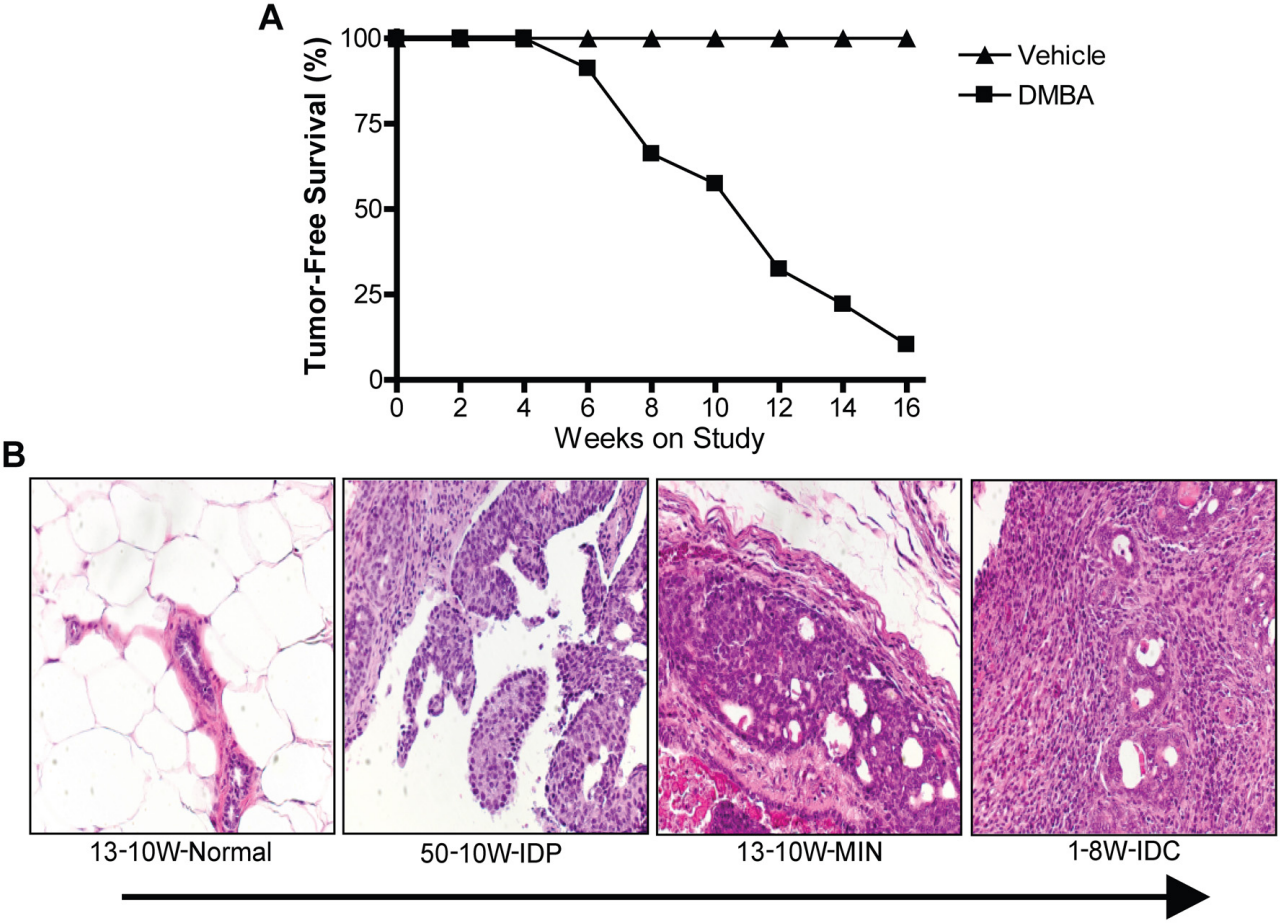
Time course study tumor development allowed collection of mammary tissue throughout progression. (A), Percentage of rats without palpable tumor in response to oral gavage of 65 mg/kg DMBA or vehicle-only treatment until study endpoint 16-weeks post administration. (B), representative H&E sections of normal mammary ductal tissue, intraductal proliferation (IDP) tissue, mammary intraepithelial neoplasia (MIN) tissue, and invasive ductal carcinoma (IDC) tissue.

To study the miRNA profiles across progression stages in a model of luminal mammary carcinoma, rats were treated with the carcinogen DMBA and euthanized every two weeks between a period of 6–16 weeks post-DMBA administration, based on when tumor was palpable. By 16-weeks post-DMBA administration, 90% of the DMBA-treated rats had developed palpable tumors in their abdominal mammary fat pads (MFPs), while no tumors developed in the vehicle-only control rats (Fig 1A). Tumor-bearing MFPs of DMBA-treated rats were scored to identify areas of tissue representing four distinct pathological characterizations, including normal, intraductal proliferation (IDP), mammary intraepithelial neoplasia (MIN), or invasive ductal carcinoma (IDC) (Fig 1B). Normal ducts were identified based on organized, single layer ductal epithelium while IDP was characterized by thickening of the ductal epithelium and the presence of nuclear atypia. MIN lesions presented highly atypical cells filling, yet confined within, the duct and IDC consisted of highly atypical cells infiltrating past the basement membrane into surrounding mammary tissue.

**Fig 2:**
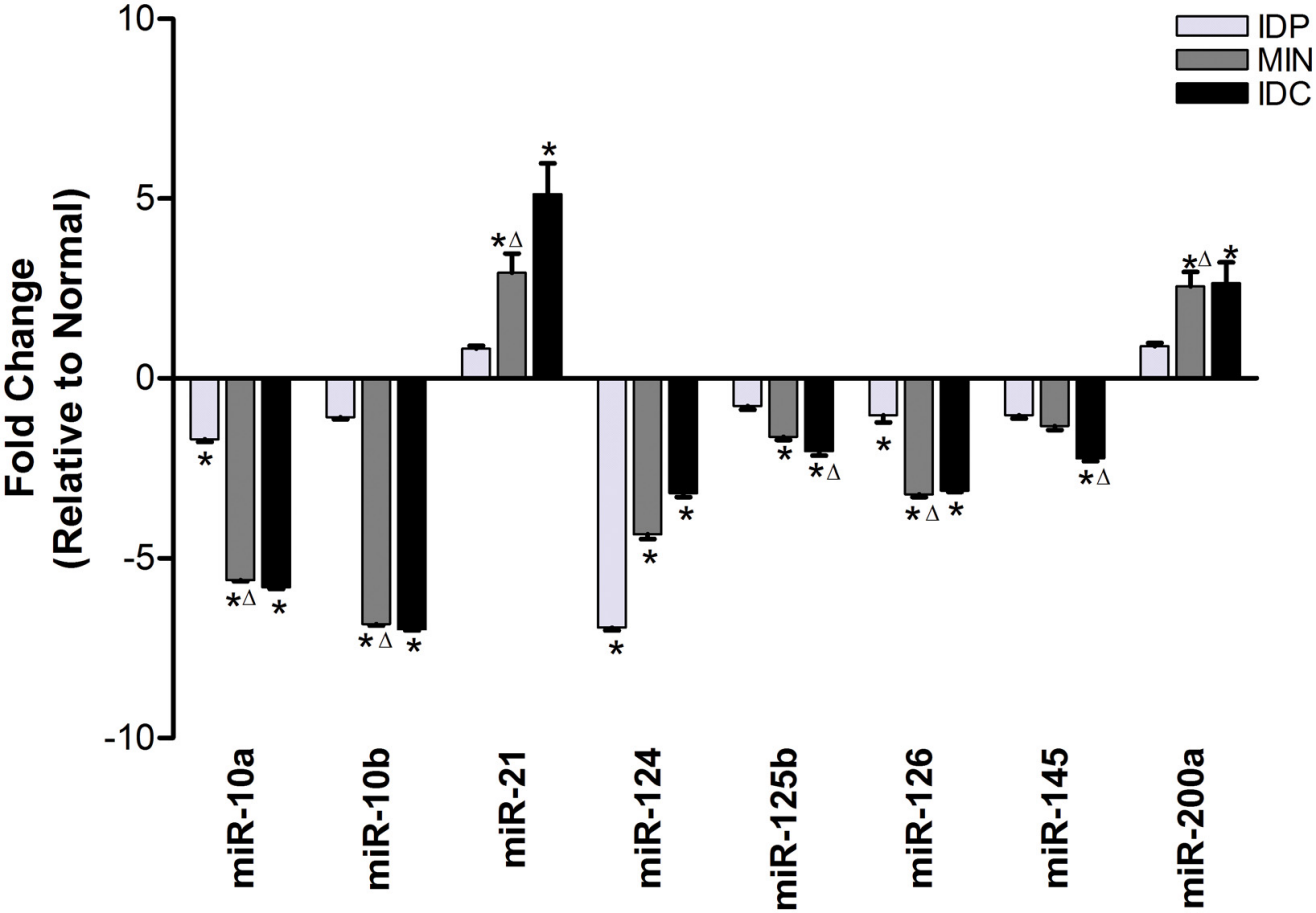
Eight miRNAs are associated with progression in DMBA-induced model of luminal mammary cancer. Real-time PCR quantification of the eight miRNAs that showed increasing dysregulation away from normal expression as tumor progression advanced. Expression of each miRNA at each progression stage is represented as a fold change relative to expression level in normal tissue, which therefore is at one and is not shown on the graph. Error bars represent standard error of the mean. *—significant difference from normal tissue expression, Δ –significant difference from previous lesion grade expression, P<0.05.

Tissue regions representing each of these four stages were microdissected for individual analysis. Global miRNA expression analysis was done on each lesion grade by microarray as a screen to identify miRNAs of interest that may be differentially expressed between progression stages (Table 1). Forty-four miRNAs were selected from those identified to be of interest and were tested by RT-qPCR using the same RNA samples used in the global array. This initial test identified 15 miRNAs that were trending toward differences in expression between any two stages. These 15 miRNAs were then subjected to more rigorous RT-qPCR testing and ultimately 8 miRNAs were identified that are increasingly dysregulated from normal expression levels as the severity of mammary lesion increases (Fig 2). The majority of these progression-associated miRNAs, including miR-10a, miR10b, miR-124, miR-125b, miR-126, miR-145, were increasingly downregulated with increasing lesion severity, while 2 miRNAs, miR-21 and miR-200a, were upregulated with advancing tumor progression.

**Table 1:** Fold change in microRNA expression between progressive stages of luminal mammary cancer development in a rodent DMBA-induced model.

microRNA	IDP vs Norm	MIN vs IDP	IDC vs MIN	microRNA	IDP vs Norm	MIN vs IDP	IDC vs MIN	microRNA	IDP vs Norm	MIN vs IDP	IDC vs MIN
hsa-miR-1	78.854	0.017	11.879	hsa-miR-152	—	—	0.438	hsa-miR-432	—	2.757	—
hsa-miR-15a	0.351	3.417	—	hsa-miR-155	5.417	—	2.681	hsa-miR-453	—	3.356	0.429
hsa-miR-17	0.332	2.761	2.896	hsa-miR158	—	2.799	—	hsa-miR-483-	—	2.960	—
hsa-miR-20b	—	—	5.829	hsa-miR160	—	2.404	—	hsa-miR-484	—	—	2.021
hsa-miR-21	0.459	—	—	hsa-miR-183	0.342	—	—	hsa-miR-492	—	—	8.364
hsa-miR-26b	—	—	2.205	hsa-miR-185	7.563	—	—	hsa-miR-501	2.992	—	—
hsa-miR-28	0.128	—	—	hsa-miR186	—	0.429	0.492	hsa-miR-503	3.940	—	—
hsa-miR-29b	0.357	3.548	—	hsa-miR-187	0.444	0.221	3.763	hsa-miR-510	—	—	2.583
hsa-miR-29c	0.217	4.043	3.885	hsa-miR-188	—	—	0.478	hsa-miR-518c	3.644	0.438	3.509
hsa-miR30	—	—	5.731	hsa-miR-193a	—	3.626	—	hsa-miR-519e	—	—	2.935
hsa-miR-30a	—	—	5.096	hsa-miR-193b	0.236	3.484	—	hsa-miR-527	—	—	2.004
hsa-miR-30b	—	3.027	—	hsa-miR-194	—	3.219	—	hsa-miR-552	0.191	—	—
hsa-miR-30c	0.354	4.523	—	hsa-miR-196a	9.077	—	0.154	hsa-miR-566	—	3.757	2.595
hsa-miR-30e	0.156	—	—	hsa-miR-200a	0.129	4.444	6.503	hsa-miR-571	6.166	—	—
hsa-miR-31	0.383	—	—	hsa-miR-200b	0.447	—	—	hsa-miR-574	—	—	0.356
hsa-miR-34a	0.262	2.198	—	hsa-miR-206	21.439	0.067	5.582	hsa-miR-575	0.309	5.327	—
hsa-miR-92b	0.029	—	—	hsa-miR208	0.48377	—	—	hsa-miR-595	—	2.260	—
hsa-miR-95	—	3.338	0.414	hsa-miR-212	—	—	0.442	hsa-miR-601	—	—	2.599
hsa-miR-98	—	—	3.556	hsa-miR-213	0.074	—	—	hsa-miR-608	0.412	—	—
hsa-miR102	3.890	—	—	hsa-miR219	—	—	2.629	hsa-miR-610	6.083	0.468	—
hsa-miR-106a	0.499	―	—	hsa-miR223	3.566	3.714	—	hsa-miR-611	0.008	—	—
hsa-miR-124a	—	6.951	0.345	hsa-miR246	4.717	—	—	hsa-miR-612	—	3.034	0.381
hsa-miR-126	—	—	0.497	hsa-miR254	—	—	2.170	hsa-miR-619	—	0.401	—
hsa-miR128	—	—	0.206	hsa-miR258	3.530	2.374	—	hsa-miR-622	—	—	0.380
hsa-miR-129	5.472	—	—	hsa-miR-324	0.111	16.217	—	hsa-miR-623	0.181	0.282	2.380
hsa-miR-130b	5.764	—	4.818	hsa-miR-328	—	—	0.053	hsa-miR-628	—	5.259	—
hsa-miR-133a	46.673	0.007	—	hsa-miR-342	—	—	5.336	hsa-miR-630	3.469	0.240	—
hsa-miR-133b	122.117	0.040	9.901	hsa-miR-362	—	—	2.820	hsa-miR-633	8.840	0.104	—
hsa-miR-134	—	—	0.467	hsa-miR-373	6.130	0.406	—	hsa-miR-636	—	—	0.172
hsa-miR-138	—	0.398	—	hsa-miR-375	—	0.493	—	hsa-miR-648	—	0.417	2.543
hsa-miR-139	0.135	—	—	hsa-miR-378	—	—	3.902	hsa-miR-650	10.095	—	0.322
hsa-miR141	3.098	—	2.076	hsa-miR-382	3.147	7.132	—	hsa-miR-658	4.005	0.392	—
hsa-miR-142	—	—	2.480	hsa-miR-421	0.439	2.380	—	hsa-miR-662	—	6.114	3.248
hsa-miR-146a	—	—	2.557	hsa-miR-425	—	2.41603	0.085				

IDP, intraductal proliferation; MIN, mammary intraepithelial neoplasia; IDC, invasive ductal carcinoma. MiRNAs identified to be of interest based on a change in expression of at least 2-fold, array readout of one group averaging above minimal acceptable limit, and all readouts within one group being above or below average readout of its comparison group.

**Fig 3:**
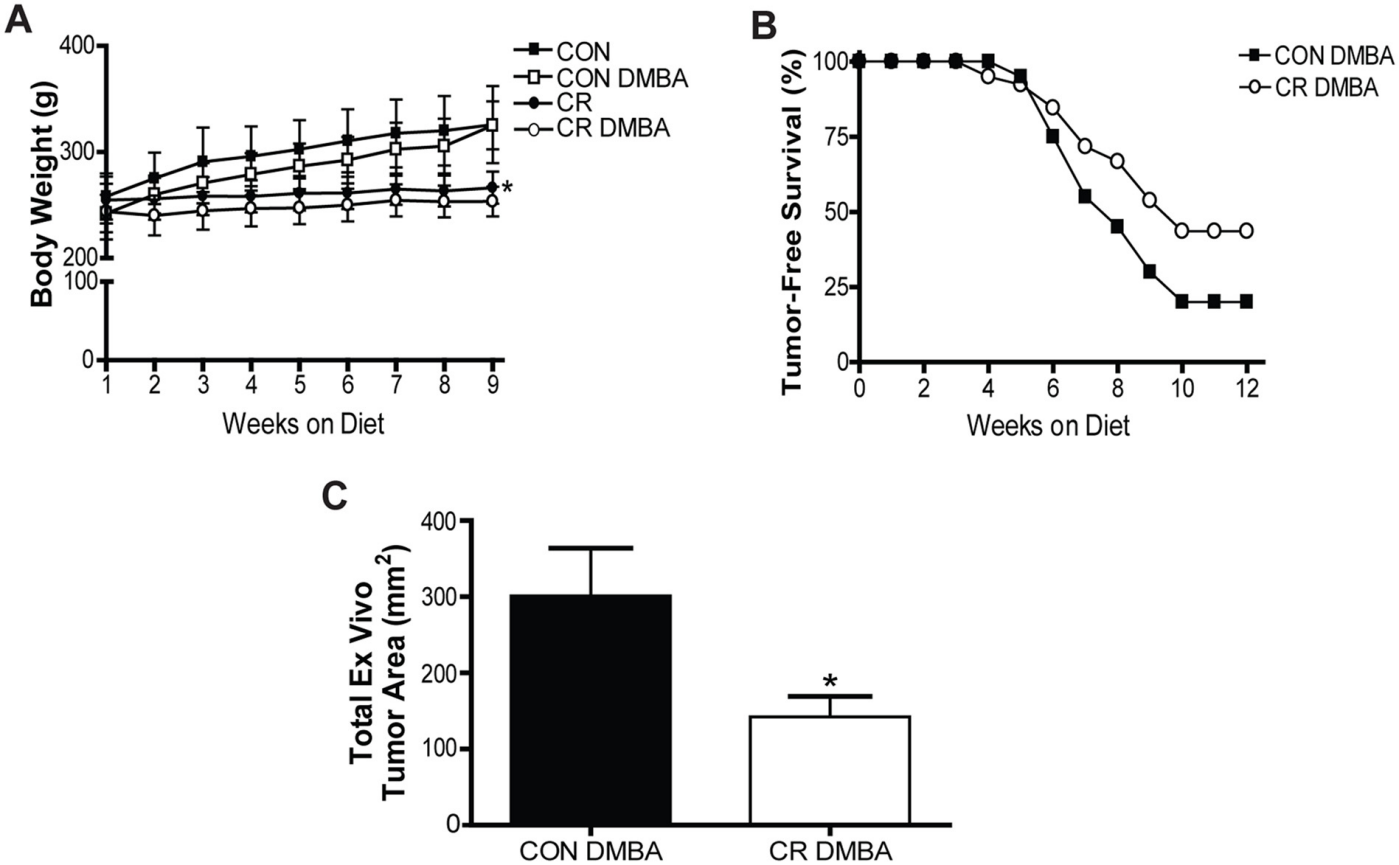
Calorie restriction increases survival and decreases tumor burden relative to control diet. (A), average body weights of rats within study groups. (B), Percentage of overall survival following DMBA treatment until study endpoint. (C), Average total *ex vivo* tumor area of rats treated with DMBA. Error bar indicate standard deviation, P<0.05.

To investigate which of these progression-associated miRNAs were modulated by CR, a second animal study was conducted involving DMBA-treated rats receiving either an *ad libitum*-fed control diet or a CR diet. Rats on the CR regimen maintained a steady body weight over the course of the study while control rats consistently gained weight, leading to a significant difference in body weight between CR rats and their Control counterparts at 9 weeks on diet, the latest point at which 50% of rats in any group remained tumor-free (P<0.001) (Fig 3A). There was no difference in body weight between DMBA-treated and vehicle-treated rats within the same diet group. After 12 weeks on diet, 20% of Control rats had not developed tumor, while 43.6% of CR rats remained tumor-free (P = 0.013) (Fig 3B). Of the tumors that did form, those from CR rats (median = 109.4mm^2^) were significantly smaller than those from the Control group (median = 250.9, P = 0.009) (Fig 3C). Taken together these results indicate an increase in tumor-free survival and a reduction in tumor burden with the CR diet.

**Fig 4:**
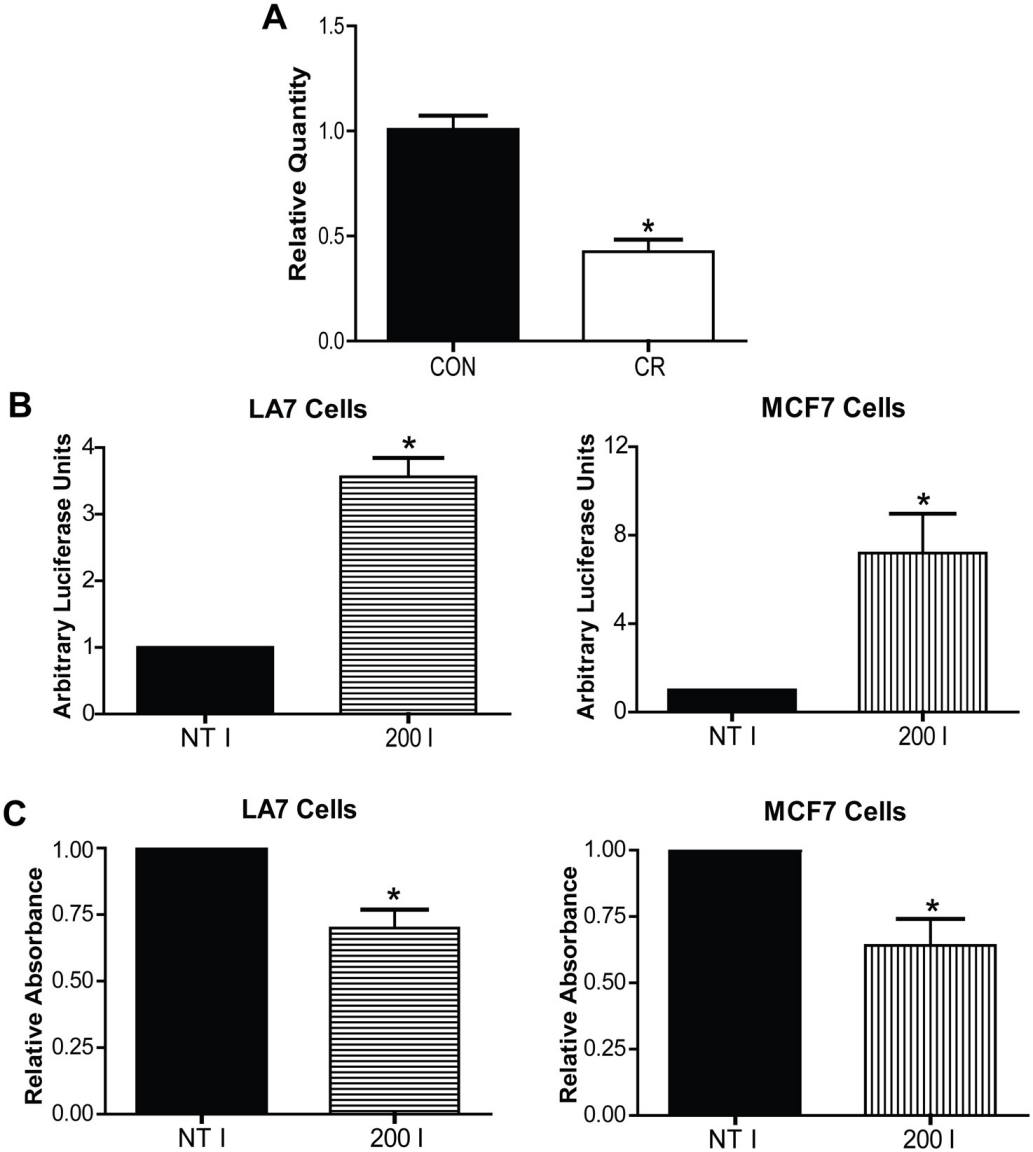
The downregulation of progression-associated miR-200a by CR inhibits cellular proliferation. (A), Real-time PCR quantification of tumoral miR-200a expression. (B), Luminescence signal following miR-200a inhibition indicating efficiency of miR-200a targeting. (C), A_370_ following BrdU incorporation indicating cellular proliferation. Error bars indicate standard error of the mean, P<0.05.

The 8 miRNAs we identified to be progression-associated were assessed in tumor RNA samples from Control and CR mice, and only one, miR-200a, was found to be CR-responsive (Fig 4A). Specifically, miR-200a was downregulated by 57% in CR tumors compared to Control tumors (P = 0.002). When miR-200a function was inhibited *in vitro* in both rat and human luminal mammary cancer cell lines to mimic the effect of CR on this miRNA, cellular proliferation was significantly decreased compared to a non-targeting inhibitor control (LA7: P = 0.012, MCF7: P = 0.037)(Fig 4B and 4C). This result indicates that miR-200a has a pro-proliferative function in mammary cancer cells and corresponds with the finding that its expression increases *in vivo* throughout progression of luminal mammary cancer.

## Discussion

The investigation of miRNAs as powerful post-transcriptional regulators has proven useful in understanding, profiling, and treating breast cancers [10]. Different subtypes of human breast cancer are known to display distinct gene expression profiles, although profiling of miRNAs associated with development or progression of ER-positive luminal cancers is lacking [14,22]. Here, we present for the first time a miRNA expression profile across the tumorigenesis spectrum, from normal to preneoplastic (IDP to MIN) and ultimately to IDC, in a well-characterized rat model of luminal mammary carcinoma. We identified 8 miRNAs that are associated with mammary tumor progression, and found that one of these, miR-200a, is associated with the potent anticancer effects of a 30% CR diet regimen.

The 8 miRNAs we found from our profiling (specifically, miR-10a, miR-10b, miR-21, miR-124, miR-125b, miR-126, miR-145, and miR-200a) each showed progressive changes in expression with advancing lesion grade. The majority of the identified miRNAs are progressively downregulated from normal expression levels, a finding that is consistent with the general understanding that miRNAs are globally lost in breast cancers [23]. However, the expression of miR-21 and miR-200a increased throughout progression, a pattern that suggests these miRNAs function as oncomiRs in this model. Several of the progression-associated miRNAs identified mirror those found to be changed during progression in human normal, DCIS, and IDC breast cancer samples, including an upregulation of miR-21 and a downregulation of miR-10b, miR-125b, and miR-126 [15]. Further, it is interesting to note in our data that the largest proportional change in miRNA expression consistently occurred between IDP and MIN, suggesting this transition is a critical window for pro-tumorigenic alterations. Insights such as this are facilitated by looking at profiles across progression stages, deepening our understanding of the biology of cancer development and miRNA involvement beyond simply looking at what is altered between normal tissue and carcinoma.

We and others have demonstrated the strong anticancer effects of CR in multiple rodent models of mammary cancer, although the effect of CR on luminal mammary tumors, the most common type of breast cancer in humans, has not been extensively characterized [5,6,24]. Here we found that 30% CR significantly increased tumor-free survival and decreased total size of the tumors that did develop. These findings are in agreement with the few other studies that have implemented CR in a luminal mammary tumor model, although the model was not characterized as such at the time [25,26,27]. This series of studies led by Kritchevsky and colleagues found that a range in degrees of CR in different dietary contexts significantly reduced tumor incidence, multiplicity, and weight, although the molecular mechanisms behind this protective effect remained largely undefined. Presently, we have shown that the regulation of miR-200a may be one such mechanism. Of the eight progression-associated miRNAs we identified, miR-200a was the only one found to be CR-responsive. While expression of this miRNA increased with advancing cancer progression in rats on the *ad libitum* Control diet, miR-200a expression was significantly downregulated in CR tumors compared to Control tumors, indicating that the CR diet maintains miR-200a expression at levels closer to those seen in normal tissue. Considering our results to this point, we hypothesized that miR-200a was acting as an oncomiR in this model and that CR’s normalization of miR-200a could be contributing to the protective effect of this dietary regimen.

Although the miR-200 family of miRNAs are most traditionally thought of as tumor-suppressive miRNAs because of their ability to target epithelial-to-mesenchymal transition-promoting transcripts, it is clear that miRNAs have many different functions through the targeting of hundreds of mRNAs, and thus different functions can be dominant in different biological settings [11,28,29]. MiR-200a is upregulated in many types of cancerous tissue including mammary cancer, and this upregulation can contribute to tumorigenesis by affecting proliferation, oxidative stress responses, and/or resistance to cell death [30,31,32,33]. Considering our finding that tumors in CR mice were significantly smaller relative to those in Control mice, we investigated how CR’s regulation of miR-200a may affect cellular proliferation. When miR-200a was inhibited in both rat and human luminal mammary cancer cell lines, mimicking CR’s effect on this miRNA, we found proliferation was significantly reduced. This finding suggests that CR may reduce tumor burden by dampening cellular proliferation through the prevention of miR-200a upregulation.

In summary, we have presented, for the first time, a luminal mammary cancer progression-associated miRNA profile and have shown that CR prevents the dysregulation of one of these miRNA, miR-200a. Further, we have shown that CR greatly reduces tumor initiation and growth, a protective effect that can be explained, at least in part, through CR’s normalization of miR-200a expression and resultant inhibition of cellular proliferation. These findings deepen our understanding of the dysregulation of miRNA throughout cancer progression and suggest that miR-200a may be a novel intervention target for mimicking the suppressive effects of CR on mammary tumor development and growth.
